# Supplementary material for: ZIC-cHILIC Functionalized Magnetic Nanoparticle for Rapid and Sensitive Glycopeptide Enrichment from <1 µL Serum
Source: Nanomaterials (Basel). 2021 Aug 24;11(9):2159. doi: 10.3390/nano11092159 (PMC8470806; doi:10.3390/nano11092159)
Supplement: Supplementary file 1 [file nanomaterials-11-02159-s001.zip › nanomaterials-1332399-supplementary.pdf]

Supplementary Materials

ZIC-cHILIC Functionalized Magnetic Nanoparticle for Rapid and Sensitive Glycopeptide Enrichment from < 1 µL Serum

Tiara Pradita [1,2,3], Yi-Ju Chen [1], Elias Gizaw Mernie [1], Sharine Noelle Bendulo [1] and Yu-Ju Chen [1,2,4]*

[1] Institute of Chemistry, Academia Sinica, Taipei 115, Taiwan; tpradita01@gate.sinica.edu.tw (T.P.); tp7249@gate.sinica.edu.tw (Y.-J.C.); eliasgizaw6@gmail.com (E.G.M.); sobendulo@gmail.com (S.N.B.)

[2] Sustainable Chemical Science and Technology, Taiwan International Graduate Program, Academia Sinica, Taipei 115, Taiwan

[3] Department of Applied Chemistry, National Yang Ming Chiao Tung University, Hsinchu 300, Taiwan

[4] Department of Chemistry, National Taiwan University, Taipei 106, Taiwan

* Correspondence: yujuchen@gate.sinica.edu.tw; Tel.: +886-2-5572-8660

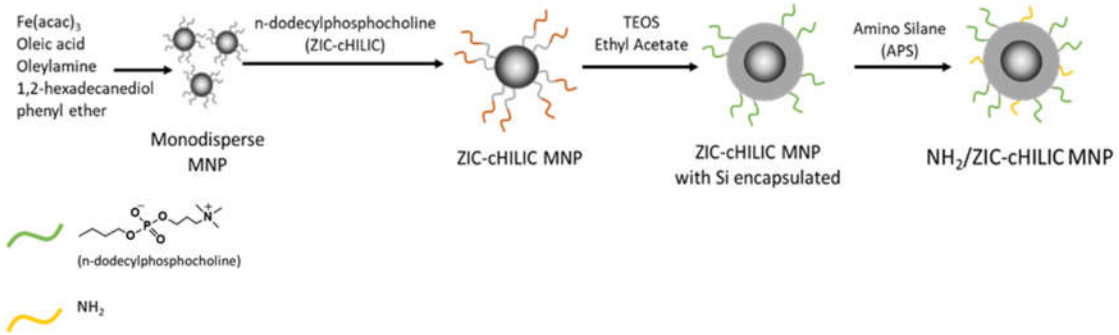

Figure S1. Synthetic route of ZIC-cHILIC@MNPs

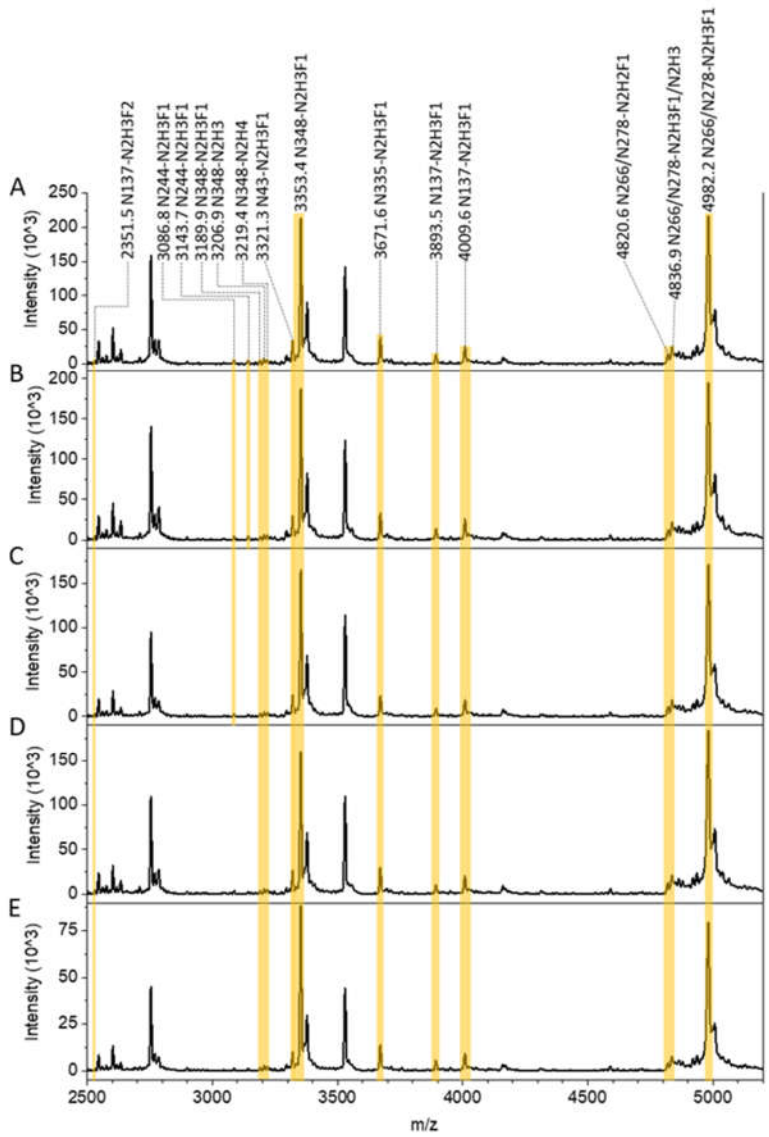

Figure S2. Reusability test of ZIC-cHILIC@MNPs for HRP glycopeptide enrichment using MALDI-TOF analysis. The MALDI-TOF spectra of 1st to 5th round of enrichment were presented in (A)–(E).

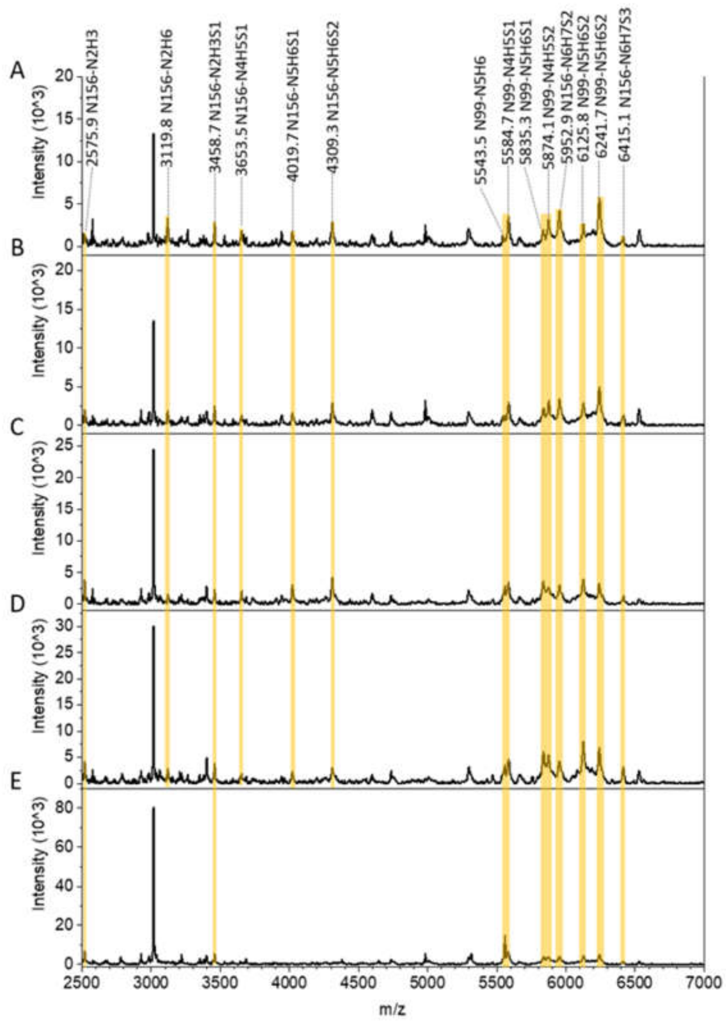

Figure S3. Reusability test of ZIC-cHILIC@MNPs for fetuin glycopeptide enrichment using MALDI-TOF analysis. The MALDI-TOF spectra of 1st to 5th round of enrichment were presented in (A)–(E).

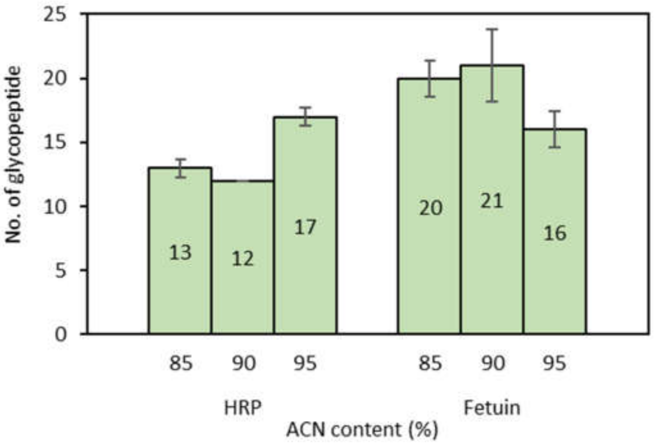

Figure S4. Effect of ACN concentration on number of enriched glycopeptides from standard HRP and fetuin using ZIC-cHILIC@MNPs.

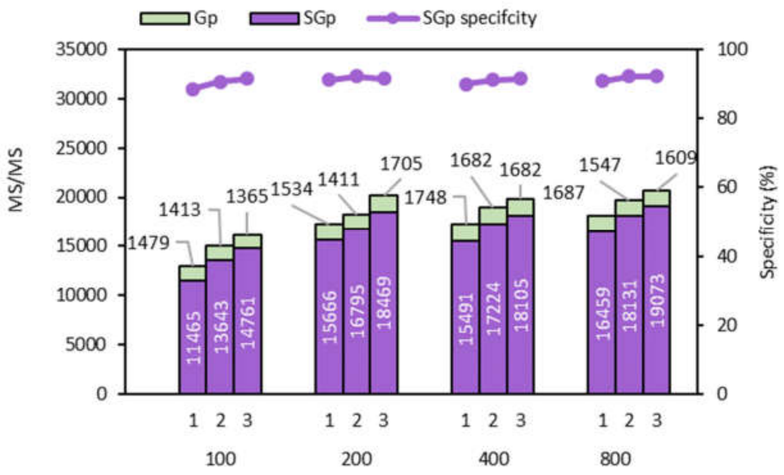

Figure S5. Number and specificity of sialylated glycopeptides towards total glycopeptides in MS/MS level from one of HCC patient serum.

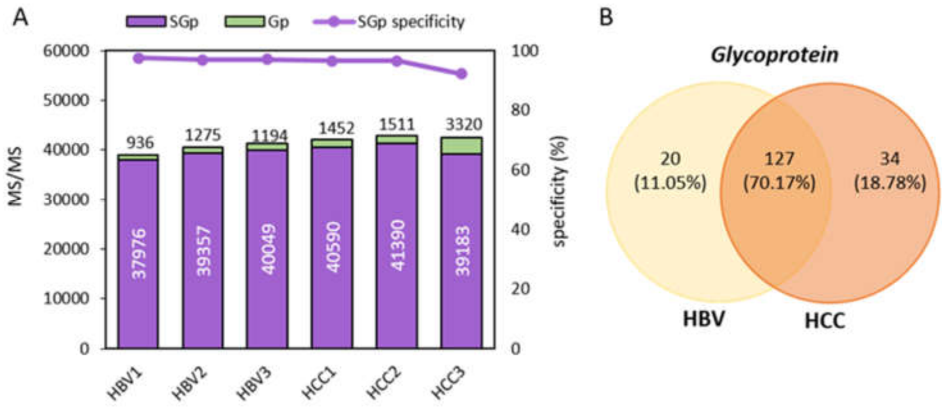

Figure S6. (**A**) Sialo-glycopeptide specificity towards total glycopeptides in MS/MS level from individual HBV (n=3) and HCC (n=3) patient serum. (**B**) Venn diagram of identified glycoproteins from two patient cohorts.

Table S1. Zeta potential measurement of core MNPs and ZIC-cHILIC@MNPs.

Material	Zeta Potential (mV)	STDEV
Fe_3O_4 Core MNPs	−1.62	±20.2
Fe_3O_4 Core MNPs	−22.20	±4.8

Table S2. List of glycopeptides enriched from HRP by using ZIC-cHILIC@MNPs.

Scheme .	m/z	Site	Motif	Peptide Sequence	Modifications	Glycan
GH1	1842.8	N234	NVG	NVGLNR		N2H3cF1
GH2	2531.5	N137	NTT	ASILLDNTTSFR	Deamidation	N2H3cF2
GH3	3086.8	N244	NLS	GLCPLNGNLSALVDFDLR		N2H3cF1
GH4	3143.7	N244	NLS	GLCPLNGNLSALVDFDLR	Carbamidometh yl	N2H3cF1
GH5	3190.6	N348	NST	SFANSTQTFFNAFVEAMDR		N2H2cF1
GH6	3207.4	N348	NST	SFANSTQTFFNAFVEAMDR		N2H3
GH7	3222.5	N348	NST	SFANSTQTFFNAFVEAMDR	Methylation	N2H4
GH8	3299.5	N238	NRS	NVGLNRSSDLVALSGGHTFGK		N2H3cF1
GH9	3321.6	N43	NVS	QLTPTFYDNSCPNVSNIVR		N2H3cF1
GH10	3353.4	N348	NST	SFANSTQTFFNAFVEAMDR		N2H3cF1
GH11	3369.5	N348	NST	SFANSTQTFFNAFVEAMDR	Oxidation (M)	N2H3cF1
GH12	3671.7	N335	NAT	GLIQSDQELFSSPNATDTIPLVR		N2H3cF1
GH13	3895.7	N137	NTT	LHFHDCFVNGCDASILLDNTTSF R		N2H3cF1
GH14	4009.7	N137	NTT	LHFHDCFVNGCDASILLDNTTSF R	2xCarbamidom ethyl	N2H3cF1
GH15	4024.7	N137	NTT	LHFHDCFVNGCDASILLDNTTSF R	2xCarbamidom ethyl	N2H3cF2
GH16	4056.0	N43	NVS	QLTPTFYDNSCAAVESACPRPNV SNIVR	-H2O	N2H3
GH17	4164.8	N137	NTT	LHFHDCFVNGCDASILLDNTTSF RTEK	Carbamidometh yl	N2H3
GH18	4821.7	N266/N278	NFS/NTT	LYNFSNTGLPDPTLNTTYLQTLR		N2H2cF1/N2H2 cF1
GH19	4837.1	N266/N278	NFS/NTT	LYNFSNTGLPDPTLNTTYLQTLR		N2H3/N2H3cF1
GH20	4983.2	N266/N278	NFS/NTT	LYNFSNTGLPDPTLNTTYLQTLR		N2H3cF1/N2H3 cF1

Table S3. List of glycopeptides enriched from fetuin by using ZIC-cHILIC@MNPs.

Scheme .	m/z	Site	Motif	Peptide Sequence	Modifications	Glycan
GF1	2576.9	N156	NDS	LCPDCPLLAPLNDSR	1xCarbamidomethyl	N2H3
GF2	3040.2	N156	NDS	LCPDCPLLAPLNDSR	2xCarbamidomethyl	N4H3
GF3	3121.4	N156	NDS	LCPDCPLLAPLNDSR	2xCarbamidomethyl	N2H6
GF4	3219.7	N176	NGS	VVHAVEVALATFNAESNGSYLQLVEIS R		N1
GF5	3459.1	N156	NDS	KLCPDCPLLAPLNDSR	2xCarbamidomethyl	N4H3S1
GF6	3653.5	N156	NDS	KLCPDCPLLAPLNDSR	2xCarbamidomethyl	N4H5S1
GF7	3686.6	N156	NDS	LCPDCPLLAPLNDSR		N4H5F1S1
GF8	3701.5	N156	NDS	LCPDCPLLAPLNDSR	2xCarbamidomethyl	N5H3S1
GF9	4019.7	N156	NDS	LCPDCPLLAPLNDSR	2xCarbamidomethyl	N5H6S1
GF10	4173.7	N156	NDS	LCPDCPLLAPLNDSR	2xCarbamidomethyl	N6H3F1S2
GF11	4309.3	N156	NDS	LCPDCPLLAPLNDSR	2xCarbamidomethyl	N5H6S2
GF12	5038.1	N156	NDS	LCPDCPLLAPLNDSR	1xCarbamidomethyl	N6H7S3
GF13	5180.3	N99	NCS	RPTGEVYDIEIDTLETTCHVLDPTPLAN CSVR		N4H5
GF14	5469.4	N99	NCS	RPTGEVYDIEIDTLETTCHVLDPTPLAN CSVR		N4H5S1
GF15	5545.8	N99	NCS	RPTGEVYDIEIDTLETTCHVLDPTPLAN CSVR		N5H6
GF16	5556.9	N156	NDS	CDSSPDSAEDVRKLCPDCPLLAPLNDS R	2xCarbamidomethyl	N5H6S1
GF17	5584.9	N99	NCS	RPTGEVYDIEIDTLETTCHVLDPTPLAN CSVR	2xCarbamidomethyl	N4H5S1
GF18	5662.4	N156	NDS	CDSSPDSAEDVRKLCPDCPLLAPLNDS R		N6H7S1
GF19	5758.9	N156	NDS	CDSSPDSAEDVRKLCPDCPLLAPLNDS R	3xCarbamidomethyl	N5H6S2
GF20	5835.8	N99	NCS	RPTGEVYDIEIDTLETTCHVLDPTPLAN CSVR		N5H6S1
GF21	5874.1	N99	NCS	RPTGEVYDIEIDTLETTCHVLDPTPLAN CSVR	2xCarbamidomethyl	N4H5S2
GF22	6048.1	N156	NDS	CDSSPDSAEDVRKLCPDCPLLAPLNDS R	3xCarbamidomethyl	N5H6S2
GF23	5951.5	N156	NDS	CDSSPDSAEDVRKLCPDCPLLAPLNDS R		N6H7S2
GF24	6124.9	N99	NCS	RPTGEVYDIEIDTLETTCHVLDPTPLAN CSVR		N5H6S2
GF25	6242	N99	NCS	RPTGEVYDIEIDTLETTCHVLDPTPLAN CSVR	2xCarbamidomethyl	N5H6S2
GF26	6413.1	N156	NDS	CDSSPDSAEDVRKLCPDCPLLAPLNDS R	3xCarbamidomethyl	N6H7S3
GF27	6534.1	N156	NDS	CDSSPDSAEDVRKLCPDCPLLAPLNDS R		N6H7S4
GF28	7190.4	N99	NCS	RPTGEVYDIEIDTLETTCHVLDPTPLAN CSVR	2xCarbamidomethyl	N6H7S4
